# Identification of Resistance Sources and Genome-Wide Association Mapping of Septoria Tritici Blotch Resistance in Spring Bread Wheat Germplasm of ICARDA

**DOI:** 10.3389/fpls.2021.600176

**Published:** 2021-05-25

**Authors:** Sara Louriki, Sajid Rehman, Samira El Hanafi, Yassine Bouhouch, Muamar Al-Jaboobi, Ahmed Amri, Allal Douira, Wuletaw Tadesse

**Affiliations:** ^1^Biodiversity and Crop Improvement Program, International Center for Agricultural Research in the Dry Areas, Rabat, Morocco; ^2^Laboratoire de Productions Végétales, Animales et Agro-industrie, Département de Biologie, Faculté des Sciences, Université Ibn Tofail, Kenitra, Morocco; ^3^Physiology Plant Biotechnology Unit, Bio-bio Center, Faculty of Sciences, Mohammed V University in Rabat, Rabat, Morocco

**Keywords:** Septoria tritici blotch, seedling resistance, adult plant resistance, spring bread wheat, GWAM, QTL

## Abstract

Septoria tritici blotch (STB) of wheat, caused by the ascomycete *Zymoseptoria tritici* (formerly *Mycosphaerella graminicola*), is one of the most important foliar diseases of wheat. In Morocco, STB is a devastating disease in temperate wheat-growing regions, and the yield losses can exceed up to 50% under favorable conditions. The aims of this study were to identify sources of resistance to STB in Septoria Association Mapping Panel (SAMP), which is composed of 377 advanced breeding lines (ABLs) from spring bread wheat breeding program of ICARDA, and to identify loci associated with resistance to STB at seedling (SRT) as well as at the adult plant (APS) stages using genome-wide association mapping (GWAM). Seedling resistance was evaluated under controlled conditions with two virulent isolates of STB (SAT-2 and 71-R3) from Morocco, whereas adult plant resistance was assessed at two hot spot locations in Morocco (Sidi Allal Tazi, Marchouch) under artificial inoculation with a mixture of STB isolates. At seedling stage, 45 and 32 ABLs were found to be resistant to 71-R3 and SAT-2 isolates of STB, respectively. At adult plant stage, 50 ABLs were found to be resistant at hot spot locations in Morocco. Furthermore, 10 genotypes showed resistance in both locations during two cropping seasons. GWAM was conducted with 9,988 SNP markers using phenotypic data for seedling and the adult plant stage. MLM model was employed in TASSEL 5 (v 5.2.53) using principal component analysis and Kinship Matrix as covariates. The GWAM analysis indicated 14 quantitative trait loci (QTL) at the seedling stage (8 for isolate SAT-2 and 6 for isolate 71-R3), while 23 QTL were detected at the adult plant stage resistance (4 at MCH-17, 16 at SAT-17, and 3 at SAT-18). SRT QTL explained together 33.3% of the phenotypic variance for seedling resistance to STB isolate SAT-2 and 28.3% for 71-R3, respectively. QTL for adult plant stage resistance explained together 13.1, 68.6, and 11.9% of the phenotypic variance for MCH-17, SAT-17, and SAT-18, respectively. Identification of STB-resistant spring bread wheat germplasm in combination with QTL detected both at SRT and APS stage will serve as an important resource in STB resistance breeding efforts.

## Introduction

Wheat is one of the most important crops produced in Morocco. On average for the period 2015–2019, the country produced 5.9 million tons (MT) of wheat grain on about 3 million hectares of land. A decline of about 37% in wheat production, from 4.1 MT in 2019 to 2.6 MT in 2020, was observed. This decline is almost 56% compared with the 5-year average due to a combination of a(biotic) stresses (FAO, 2020).

During the growing season, wheat is exposed to various fungal diseases that can cause significant yield losses. Septoria tritici blotch (STB) caused by the hemibiotroph ascomycete fungus *Zymoseptoria tritici* (teleomorph *Mycosphaerella graminicola*) is a major disease of wheat in all wheat-growing areas of the world (Sánchez-Vallet et al., 2015) and can reduce yield by 30–50% under conducive weather conditions (Eyal et al., 1987). In Morocco, STB is a serious problem in temperate rainfall wheat-growing regions, which may cause up to 40% yield losses (Ezzahiri, 2001). Host plant resistance and the use of fungicides are used to limit STB-inflicted yield losses. However, the use of pesticides is not environment friendly and fungicide resistance has been reported to diverse classes of fungicides worldwide due to evolution of insensitivity in STB populations (Cools and Fraaije, 2013; Torriani et al., 2015). Thus, breeding for disease resistance remains the most economical, effective, and environment-friendly strategy to combat STB (Steiner et al., 2017).

In STB, two types of resistances have been described. Qualitative or race-specific resistance is controlled by major genes (McCartney et al., 2002), whereas quantitative resistance or non–race-specific resistance is conferred by a large number of quantitative trait loci (QTL) with small effects (Karisto et al., 2017). To date, 21 STB resistance genes (*R* genes) and 167 QTL have been identified. These genes were identified and mapped by inoculation of a segregating population derived from a cross between a resistant and a susceptible parent with a pure isolate of *Z. tritici* under controlled conditions (Brown et al., 2015). For a durable resistance, however, wheat germplasm, either with new sources of resistance from the gene banks or carrying a combination of existing *R* genes effective at seedling resistance test (SRT) as well as at the adult plant (APS) stage, are required to provide the genetic variability for improvement of the resistance breeding programs against rapidly evolving virulent STB populations.

There are two main approaches to map quantitative trait loci: linkage/QTL mapping (bi-parental) and linkage disequilibrium (LD)/association mapping. QTL mapping requires crosses between two parents with contrasting phenotype for a particular trait of interest. By contrast, LD mapping takes advantage of past recombination events that have occurred in previous generations (Hartwell et al., 2017). It has three advantages when compared with bi-parental mapping: it has an increased resolution for mapping QTL, greater diversity of alleles can be detected, and it is much faster and efficient (Yu and Buckler, 2006). Genome-wide association mapping (GWAM) is an effective statistical approach for evaluating the strength of the association between any phenotypic trait and a marker locus (George and Cavanagh, 2015). It has been widely used to identify QTL for various traits in wheat such as root length (Ayalew et al., 2018), resistance to foliar diseases (Juliana et al., 2015; Gerard et al., 2017; Kidane et al., 2017; Ayana et al., 2018; Li et al., 2019; Yates et al., 2019), grain yield (Ward et al., 2019), coleoptile length (Li et al., 2017), and heat tolerance (Maulana et al., 2018; Tadesse et al., 2019).

The main objectives of the present study were to assess a panel of spring bread wheat composed of 377 advanced breeding lines (ABLs) for seedling (SRT) and the APS resistance, and to identify genetic loci linked to STB resistance at SRT and APS stages using GWAM.

## Materials and Methods

### Plant Material

The Septoria Association Mapping Panel (SAMP) was composed of 377 spring bread wheat ABLs from spring bread wheat breeding program of ICARDA. It included 56 synthetic hexaploid wheat lines and 321 elite lines (Supplementary Table 1). The mean heading days (HD) of 91 ± 4.0 was recorded. SAMP was evaluated for resistance to STB at seedling as well as at the adult plant stages.

### Pathogen Isolates

Based on the race analysis of 21 pure isolates of STB, collected from different agro-ecological zones of Morocco, 17 pathotypes were revealed (Louriki et al., unpublished data). Two STB isolates with wide virulence spectrum, namely, SAT-2 and 71-R3, were used for SRT screening in the greenhouse, whereas for APS, inoculum composed of a mixture of 13 STB isolates (including 71-R3 and SAT-2) was used for artificial inoculation in the field (Supplementary Table 2).

Single conidial STB isolates were maintained as lyophilized agar plugs at −80°C. For SRT, pure isolates were multiplied on YMDA (4 g yeast extract (Biokar Diagnostics, Beauvais, France), 4 g malt extract (Biokar Diagnostics), 4 g sucrose (Panreac, Barcelona, Spain), 15 g agar (Biokar Diagnostics) in 1 L distilled water) plates at 20 ± 1°C, and for field screening 13 STB isolates were multiplied in YMD broth in a shaking incubator at 120 rpm at 20 ± 1°C. Inoculum was prepared by rubbing the agar surface of 7 to 8-day-old YMDA plates with a sterile glass slide followed by filtration through a double layer of cheese cloth. However, 5 to 6-day-old liquid STB culture was used for field screening. The spore density was adjusted to 10^8^ conidia/ml with a hemocytometer (Hausser Scientific, Horsham, PA, United States) and a surfactant “Tween 20” (Sigma-Aldrich, St. Louis, MO, United States) was added to a final concentration of 0.01%.

### Seedling Resistance Test

For each ABL, 4–5 seeds in three replications were sown in peat moss supplemented with 14-14-14 NPK in 98-Ray Leach cone-tainers, with each cone having 3.8 cm diameter and 14 cm depth (Stuewe & Sons, Inc., OR, United States), in the growth chamber (Model MC1750; Snijder Scientific, Tilberg, Netherlands) with a photoperiod of 16 h light/8 h dark at 18–20 ± 1°C. The growth chamber was fitted with 54W T5 color 840 LED lights and provided light intensity of 1,200 μmol m^–2^ s^–1^. Two-week-old seedlings were inoculated with 100 ml of spore suspension (supplemented with the surfactant Tween 20 to a final concentration of 0.01%) with a hand-held sprayer until run-off followed by incubation in a humid chamber with 12 h dark at 20°C/12 h night at 18 ± 1°C for 72 h to enhance spore germination and infection process. Then the seedlings were moved to a greenhouse with a photoperiod of 16 h light/8 h dark at 20 ± 1°C for the rest of the assay. After 21 days post-inoculation (dpi), percentage necrotic leaf area covered with pycnidia of the second leaf was estimated using 0–5 scale as described by Adhikari et al. (2003), where 0 = no sporulation; 1 = occasional pycnidia in few lesions (≤5%); 2 = low density of pycnidia in many or most lesions, usually unevenly distributed (6–15%); 3 = an even distribution of pycnidia at moderate density over most of the lesions (16–25%); 4 = a high density of pycnidia distributed over most lesions (>25%); and 5 = maximum pycnidial density (>40%). The disease scores of 0–1 classified the genotypes into resistant (R), 2 as moderately resistant (MR), 3 as moderately susceptible (MS), and 4–5 as susceptible (S).

### Adult Plant Stage Screening

Adult plant stage screening of the ABLs was carried out in the field at two INRA research stations in Morocco, Marchouch (MCH; 33°56 N, 6°63 W) and Sidi Allal Tazi (SAT; 34°52 N, 6°31 W), during 2016–2017 (SAT-17, MCH-17) and 2017–2018 (SAT-18) cropping seasons. The monthly average temperature, precipitation, and humidity during the cropping season of 2017 and 2018 in Sidi Allal Tazi has been included in Supplementary Table 5. Generally, the average precipitation between December and May was higher in 2018 (53.50 ± 36.05 mm) as compared with 2017 (27.26.50 ± 16.63 mm). In addition, the average temperature from December to May in 2018 was relatively cooler (13.94 ± 2.60°C) than in 2017 (15.85 ± 3.53°C) with a difference of 2°C.

The trial was laid out in an augmented design with four blocks where a paired row of 1 m per ABL was planted. Two susceptible checks (Achtar and Marchouch) were planted after every 20 test genotypes and each block was surrounded by a perpendicular spreader row composed of a mixture of susceptible checks to maintain a high disease pressure. Field inoculations were performed three times with knapsack sprayer with an interval of 10–15 days starting from Zadoks scale GS 30 using inoculum composed of a mixture of 13-STB isolates (Supplementary Table 2). The infection process and the disease establishment were further favored by overhead sprinklers. STB severity was evaluated on fully extended flag leaves with necrotic lesions bearing pycnidia at GS 73–75 (Tian et al., 2005) using Saari and Prescott scale (0–9) (Saari and Prescott, 1975) as reported by Osman et al. (2016) and Kidane et al. (2017). Data were recorded twice with an interval of 9 and 12 days in SAT-17 and SAT-18, respectively. However, a single observation was recorded for MCH-17. Genotypes were classified in the following categories: resistant (0–2), moderately resistant (3–4), moderately susceptible (5–6), and susceptible (7–9). The area under the disease progress curve (AUDPC) was calculated using the following equation as described by Jeger and Viljanen-Rollinson (2001):

$$\text{AUDPC}=\sum_{i=1}^{n}\left[\left(STB_{i+1}+STB_{i}\right)/2\right]\left[\left(t_{i+1}-t_{i}\right)\right]$$

where *STB*_*i*_ = STB severity on *i*^th^ days, *t*_*i*_ = time in days at *i*^th^ observation, and *n* is the total number of observations.

### Statistical Analysis

Data analysis was performed using R.^1^ Variability in disease severity was subjected to ANOVA using the mixed linear model (MLM) and significant means were compared using least significant difference (LSD). Phenotypic and genotypic coefficients of variation as well as the broad-sense heritability were computed using the following formula as described by Singh and Chaudhary (1985):

$$H^{2}=\frac{\delta g}{\delta p+\delta residual}=\frac{\delta g}{\delta g+\frac{\delta g*\delta env+\delta g*\delta year}{2}+\frac{\delta residual}{4}}$$

where δg = genotypic variance, δp = phenotypic variance, and δresidual = residual variance.

### Genotyping, Population Structure, and Linkage Disequilibrium

Septoria Association Mapping Panel was genotyped with 15K SNP array from TraitGenetics containing 12,905 functional SNP markers.^2^ After quality control, 10,173 SNP markers were found to be informative and were used for further analysis. The genetic structure of 369 genotypes was investigated using 477 unlinked SNP markers which were at least 6 cM apart and were distributed across the wheat genome. The admixture model, implemented in the STRUCTURE software (version 2.3.4) (Pritchard et al., 2000; Falush et al., 2003), was run for a burn-in period of 100,000 cycles. The posterior probabilities were estimated using the Markov Chain Monte Carlo (MCMC) with 100,000 repetitions. The number of clusters (*K*) ranged from 2 to 7, with five replications for each *K*. The Δk, implemented in the STRUCTURE HARVESTER (Evanno et al., 2005), was used to predict the appropriate number of sub-populations (Earl and vonHoldt, 2012). In addition, the results were confirmed by discrimination analysis of principal components (DAPC) using the *adegenet* package (Jombart et al., 2010) for R software and the optimal number of *K* was selected based on the lowest Bayesian Information Criterion (BIC) value.

Linkage disequilibrium was performed with TASSEL software version 5.2.53 (Bradbury et al., 2007) using SNP markers with known chromosomal position only. LD was estimated as squared allele frequency correlations (*R*^2^) and each pair of loci with *p* value of ≤0.01 was considered as significant.

### Genome-Wide Association Mapping

Genome-wide association mapping was performed with the TASSEL software (v 5.2.53) using 10,059 SNP markers with both General Linear Model (GLM) and Mixed Linear Model (MLM). SNP markers with minor allele frequency (MAF) of <5 and >10% missing data were discarded from the analysis. Two different models of GLM [GLM + population structure (Q), and GLM + PCA] and MLM [MLM + Kinship (K) + Q, and MLM + K + PCA] were used to investigate the best fitting model for the current study (Supplementary Figures 1, 2). Marker alleles with *p* value ≤0.001 in both MLM-PCA and MLM-Q models with kinship (K) were declared significantly associated with STB resistance. To minimize false positives, an adjusted *p* value was calculated using false discovery rate (FDR) approach. The physical positions of the significant markers were determined by built in BLAST search tool of Wheat@URGI portal^3^ using marker sequences against the IWGSC RefSeq v1.0 genome (Alaux et al., 2018). The genetic linkage maps were drawn using MapChart software (v 2.32) (Voorrips, 2002).

### Putative Candidate Gene Analysis

The SNP markers at the QTL peaks were used to identify candidate genes (CG) in the genomic regions encompassing the QTL in the PGSB database; the search was focused mainly on domains or genes functionally related to disease resistance mechanism. The SNP marker sequences were queried against the wheat reference genome sequence IWGSC (RefSeq v1.0) in Ensembl Plants online BLASTN search tool,^4^ and the CG were selected based on the chromosomal location, sequence identity (90–100%), higher BIT score (>180), and lower expect value (0–1e^–40^). Functional annotation of the putative CGs harboring significant SNPs was retrieved from https://triticeaetoolbox.org/wheat/genes/ and http://www.ebi.ac.uk/interpro/.

## Results

### Response of Wheat Genotypes to STB at Seedling Stage

**FIGURE 1:**
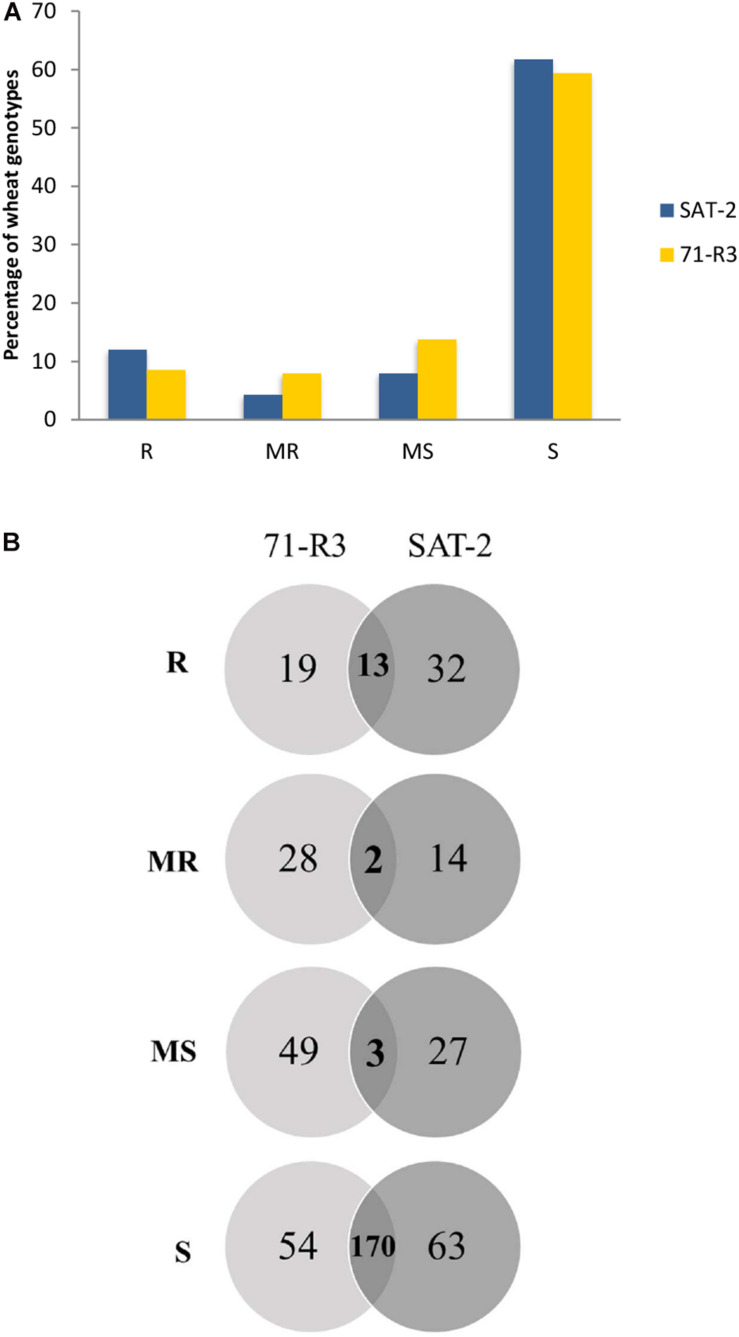
Frequency distribution of 377 advanced breeding lines (ABLs) of spring bread wheat ABLs to two Septoria tritici blotch isolates SAT-2 and 71-R3 **(A)**. Venn diagram of infection responses of 377 ABLs to two Septoria tritici blotch isolates under controlled conditions from Morocco **(B)**. Here R, MR, MS, and S represent resistant, moderately resistant, moderately susceptible, and susceptible reaction types, respectively.

Seedling screening of 377 ABLs included in the SAMP with two STB isolates (SAT-2 and 71-R3) resulted in a wide range of infection types (Supplementary Table 3). Of the 377 ABLs tested, only 45 (11.9%) and 32 (8.5%) ABLs were found to be resistant, while 16 (4.2%) and 30 (8%) of ABLs were found to be moderately resistant against SAT-2 and 71-R3 STB isolates, respectively. In contrast, 233 (61.8%) and 224 (59.4%) of ABLs were susceptible to SAT-2 and 71-R3 isolates, respectively. The infection responses of wheat genotypes to both STB isolates were skewed toward susceptibility (Figure 1 and Supplementary Table 4).

Seventeen ABLs were resistant to SAT-2 but susceptible to 71-R3 isolate, while nine ABLs were highly resistant to 71-R3 but susceptible to SAT-2 isolate, and 13 ABLs exhibited resistance at seedling stage to both STB isolates tested.

### Field Response of Wheat Genotypes to STB

**FIGURE 2:**
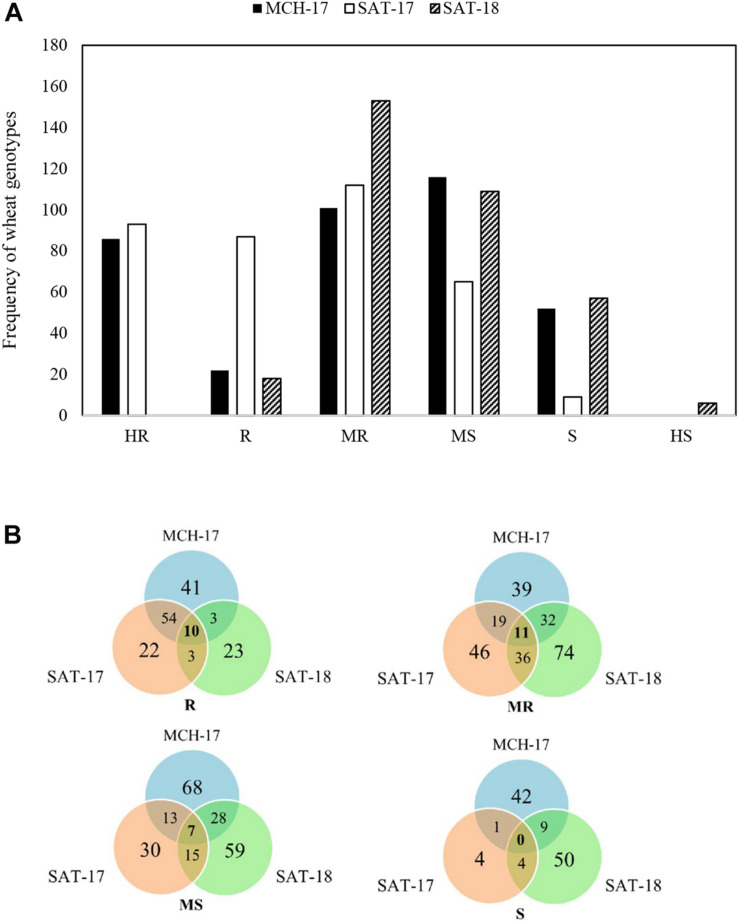
Frequency distribution of area under the disease progress curve (AUDPC) of Septoria tritici blotch in Sidi Allal Tazi during 2017 (SAT-17), 2018 (SAT-18), and in Marchouch during 2017 (MCH-17) in Morocco **(A)**. Venn diagram of field responses of 377 ABLs to Septoria tritici blotch in Sidi Allal Tazi and Marchouch in Morocco **(B)**. Here HR, R, MR, MS, S, and HS represent highly resistant, resistant, moderately resistant, moderately susceptible, susceptible, and highly susceptible, respectively.

At the adult plant stage, variations in field reactions between SAT-17, SAT-18, as well as with MCH-17 were observed (Supplementary Tables 3, 4). Of the 377 ABLs tested, 177 (46.9%) and 41 (10.8%) ABLs were found to be resistant at SAT-17 and SAT-18, respectively, however, 108 (28.6%) ABLs were resistant at MCH-17. In contrast, 52 (13.8%) ABLs were susceptible at MCH-17, while at SAT-17 and SAT-18 only 11 (2.9%) and 34 (9%) ABLs exhibited susceptibility, respectively (Figure 2 and Supplementary Tables 3, 4). Interestingly, 10 ABLs showed resistance at both locations (SAT and MCH) during 2016–2017 (SAT-17, MCH-17) and 2017–2018 (SAT-18) (Table 1). In addition, one line, namely SAMP-275 (WORRAKATTA/PASTOR//ACSAD-981), showed resistance at seedling as well as at the adult plant stage. Furthermore, four genotypes, namely SAMP-164 (WHEATEAR/22SAWSN-156), SAMP-231 (KOUKAB-1//PFAU/MILAN/3/SOSSI-3), SAMP-248 (SERI.1B^∗^2/3/KAUZ^∗^2/BOW//KAUZ/4/FLAG-1/5/WBLL1^∗^2/BRAMBLING), and SAMP-267 (FLAG-3/HIDDAB), exhibited resistance to both STB isolates at seedling and at adult plant stage at SAT-17 and MCH-17 but were moderately resistant in SAT-18.

**TABLE 1:** List of resistant (R) advance breeding lines from Septoria Association Mapping Panel (SAMP) against Septoria tritici blotch at Sidi Allal Tazi during 2017 (SAT-17), 2018 (SAT-18), and at Marchouch during 2017 (MCH-17) in Morocco.

Panel ID	Pedigree	Synthetic/elite	MCH-17	SAT-17	SAT-18
108	HUW 234/REBWAH-19	Elite	1	1	2
205	DAJAJ-5/4/CHEN/AEGILOPS SQUARROSA (TAUS)//BCN/3/KAUZ	Synthetic	1	2	2
247	FARIS-17//PFAU/MILAN/3/SOSSI-3	Elite	1	2	2
262	ZERBA-6/FLAG-6/3/TAM200/PASTOR//TOBA97	Elite	2	1	2
275	WORRAKATTA/PASTOR//ACSAD- 981	Elite	1	2	2
283	22SAWSN-142/3/PASTOR//MUNIA/ALTAR 84/4/SHAMISS-3	Synthetic	1	2	2
304	PAVON 7S3, +LR47/4/NS732/HER//ARRIHANE/3/REGRAG-1	Elite	1	2	2
305	PBW343/FLAG-4//QADANFER-4	Elite	1	1	2
350	ZERBA-6/FLAG-6/3/TAM200/PASTOR//TOBA97	Elite	1	1	2
356	FLAG-3/HIDDAB	Elite	1	1	2

The ANOVA indicated a highly significant variation (*p* < 0.001) among genotypes (G), environment × year interaction (E × Y), as well as for genotype × environment (G × E) interaction, and a significant variation (*p* < 0.05) for environment. The heritability estimate for STB was estimated to be 0.74 (Table 2).

**TABLE 2:** ANOVA for Septoria tritici blotch disease resistance in Septoria Association Mapping Panel (SAMP).

	Sum Sq	*df*	*F* value	*P* value	Heritability
Environment (E)	6.61	1	4.193	0.04097*	0.74
Genotype (G)	3,044.9	368	5.2523	<2e-16***	
E × Year (Y)	558.4	2	177.2319	<2e-16***	
G × E	1,720.95	364	3.0012	<2e-16***	
Residuals	1,099.6	698			

*Significant at the 0.05 probability level. ***Significant at the 0.001 probability level. df, degree of freedom; Sq, sum of squares.

### Marker Distribution, Population Structure, and LD

For the analysis, STB phenotypic data and 10,173 polymorphic mapped SNP markers were used. In total, the number of SNP markers assigned to the A, B, and D genomes were 3,812 (37%), 5,165 (51%), and 1,196 (12%), respectively, with B genome containing the highest while the D genome had the lowest number of markers.

**FIGURE 3:**
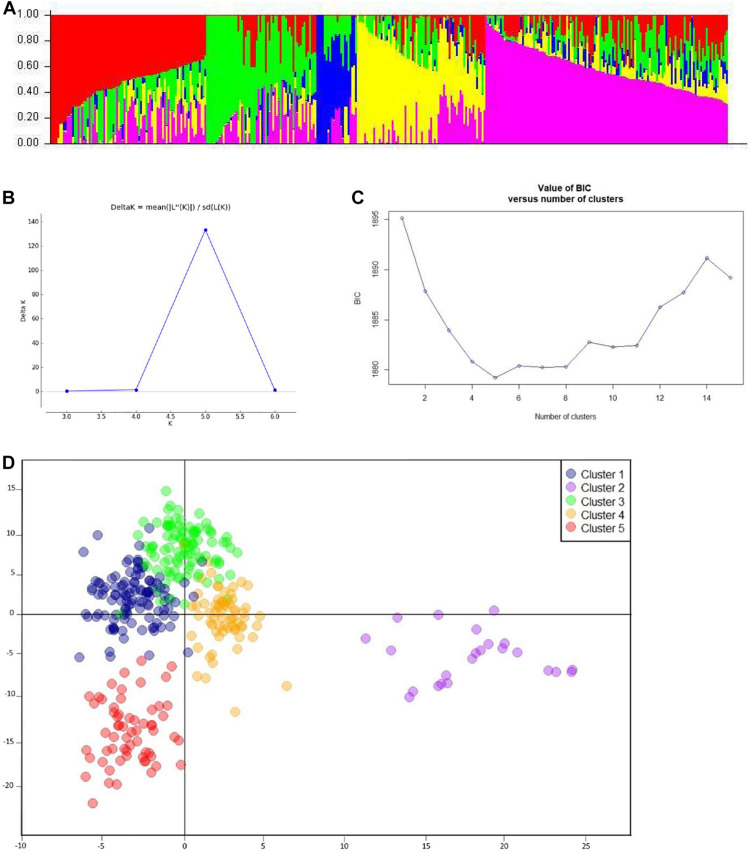
Analysis of population structure. **(A)** Estimated population structure (*K* = 5) of SAMP (369 ABLs), where each color represents a single sub-population. **(B)** Delta-k values as described by Evanno et al. (2005). **(C)** BIC plot displaying number of subpopulations/clusters. **(D)** Discriminant analysis of principal components (DAPC) for 369 wheat ABLs in SAMP.

**FIGURE 4:**
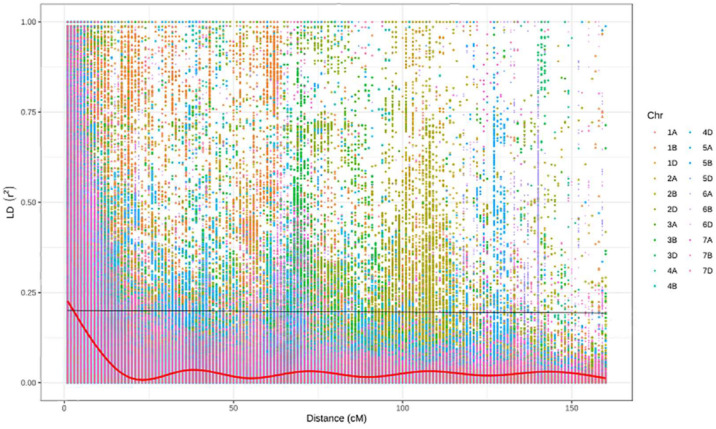
Decline of LD as measured by R^2^ against genetic distance (cM).

After filtration, a set of 9,988 SNPs was retained and used to estimate population structure, LD, and to perform GWAM. The population structure analysis divided the SAMP panel into five subpopulations (*K = 5*) (Figure 3A). The number of clusters (*K*) was plotted against the calculated Δk obtained from the STRUCTURE software. The Δk peaked clearly at *K = 5*, providing further evidence for the existence of five genetically distinct subpopulations in this association mapping panel (Figure 3B). This result was confirmed further with the lowest BIC value (Figure 3C) using *find cluster* function and DAPC analysis in R by clustering the 369 ABLs into five clear subgroups (Figure 3D). Cluster 1 was the largest with 115 genotypes accounting for 31.2% of the total genotypes. Clusters 3, 4, and 5 consisted of 102 (27.7%), 68 (18.4%), and 61 (16.5%) genotypes, respectively. However, cluster 2 was the smallest but distinct with only 23 (6.2%) genotypes. Wheat genotypes included in cluster 2 were derived from elite spring × elite winter facultative crosses (VEE/NAC, DEZ, S78-18, Cham6, Haama4). Most of the STB resistant lines were placed in subpopulation 1. In addition, a scatter plot of LD values (R^2^) was plotted against genetic distance (cM) to estimate the genome-wide LD (Figure 4), indicating a clear LD decay at 3.3 cM (*R*^2^ = 0.2).

### QTL Associated With STB Resistance

In the association analysis in TASSEL (v 5.2.53), MLM was the most suitable model both for SRT and APS data for all locations. The Q-Q and Manhattan plots for GWAM are available as Supplementary Material (Supplementary Figures 1–3).

**FIGURE 5:**
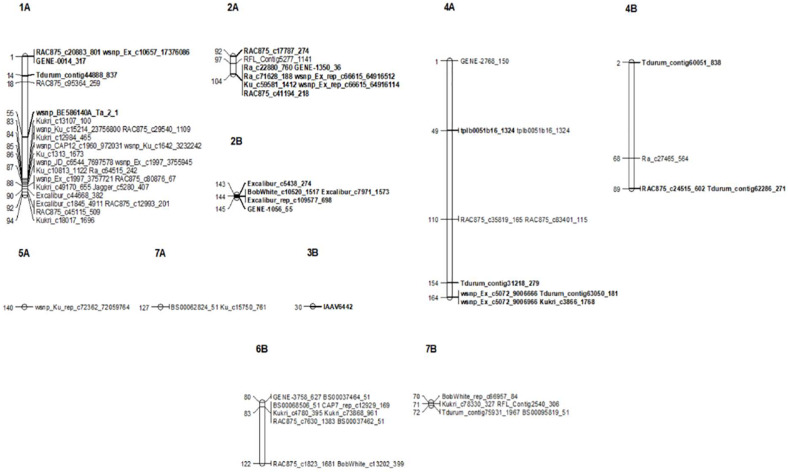
Genetic linkage map of significant SNP markers associated with seedling (SRT) and the adult plant stage (APS) resistance to STB. Markers are shown on the right and genetic distances (cM) are shown on the left. Markers in *bold* represent markers detected at SRT.

In SRT, 14 QTL, 8 for STB isolate SAT-2 and 6 for STB isolate 71-R3, were detected (Table 3 and Figure 5). For STB isolate SAT-2, QTL were detected on chromosomes 1A, 2A, 4A, 3B, and 4B with IAAV6442 being the most significant SNP marker with the highest LOD score of 3.99 on chromosome 3B. For 71-R3, QTL were detected on chromosomes 1A, 4A, 2B, and 4B with BobWhite_c10520_1517 being the most significant SNP marker with the highest LOD score of 5.19 on chromosome 2B. In total, SNP markers explained a total phenotypic variation of 33.3 and 28.3% for SAT-2 and 71-R3 isolate, respectively.

**TABLE 3:** GWAM for seedling resistance against two virulent isolates of Septoria tritici blotch (71-R3, SAT2) in Morocco.

Putative QTL	Marker	Chr.	Pos (cM)	Pos (Mb)	−log10 (*p*)	FDR*	Marker (R^2^)	Allele effect	IWGSC gene ID	Functional annotation
**71-R3**										
*SRT_71-R3_1*	wsnp_BE586140A_Ta_2_1	1A	55	31.78	4.31	0.0004	5.47	C (−13.68)	TraesCS1A02G049700	–
*SRT_71-R3_2*	Excalibur_c5438_274	2B	143	774.96	3.2	0.0008	3.85	C (8.80)	TraesCS2B02G587900	Oligosaccharyl transferase, STT3 subunit
*SRT_71-R3_3*	BobWhite_c10520_1517	2B	144	775.37	5.19	0.0004	6.66	A (10.94)	TraesCS2B02G588900	Dihydroorotate dehydrogenase, class 2
*SRT_71-R3_4*	GENE-1056_55	2B	145	779.34	3.1	0.0009	3.64	A (7.74)	TraesCS2B02G595200	Smr domain-containing protein
*SRT_71-R3_5*	tplb0051b16_1324	4A	49	473.69	3.49	0.0006	4.21	A (9.65)	TraesCS4A02G192700	–
*SRT_71-R3_6*	Tdurum_contig60051_838	4B	2	644.68	3.63	0.0006	4.45	A (−12.14)	TraesCS4B02G352700	Pyridoxal phosphate-dependent transferase
**SAT2**										
*SRT_SAT2_1*	RAC875_c20883_801	1A	1	1.21	3.5	0.0006	4.16	A (11.66)	–	–
*SRT_SAT2_2*	Tdurum_contig44888_837	1A	14	1.34	3.36	0.0006	4.03	C (−9.42)	–	–
*SRT_SAT2_3*	RAC875_c17787_274	2A	92	57.83	3.12	0.0009	3.59	C (−9.39)	–	–
*SRT_SAT2_4*	Ra_c22880_760	2A	104	498.11	3.69	0.0006	4.5	A (13.07)	TraesCS2A02G289800	Alpha-amylase inhibitors
*SRT_SAT2_5*	IAAV6442	3B	30	16.44	3.99	0.0005	5.06	A (10.07)	TraesCS3B02G034400	WD40-repeat-containing domain
*SRT_SAT2_6*	Tdurum_contig31218_279	4A	154	743.95	3.18	0.0008	3.83	A (−13.60)	–	–
*SRT_SAT2_7*	wsnp_Ex_c5072_9006666	4A	164	742.57	3.46	0.0006	4.18	A (−14.08)	TraesCS4A02G495700	SAM-dependent MTase DRM-type
*SRT_SAT2_8*	RAC875_c24515_602	4B	89	645.3	3.29	0.0007	3.91	A (9.31)	–	–

Chr, chromosome; Pos, position in cM and Mb. *FDR-adjusted p value (<0.05).

In APS, GWAM analysis identified 23 QTL using phenotypic data of SAT-17, SAT-18, and MCH-17 (Table 4 and Figure 5). The phenotypic variance explained by QTL detected for APS was 13.1, 68.6, and 11.9% for MCH-17, SAT-17, and SAT-18, respectively. The marker *R*^2^ ranged from 3.13 to 5.1%. A SNP marker, namely tplb0051b16_1324 located on chromosome 4A at 49 cM, overlapped for APS (MCH-17) as well as for SRT for isolate 71-R3. No QTL was stable across the environment tested.

**TABLE 4:** GWAM for adult plant resistance based on disease severity and AUDPC at Sidi Allal Tazi during 2017 (SAT-17), 2018 (SAT-18), and at Marchouch during 2017 (MCH-17) in Morocco.

Putative QTL	Marker	Chr.	Pos (cM)	Pos (Mb)	−log10 (*p*)	FDR*	Marker (R^2^)	Allele effect	IWGSC gene ID	Functional annotation
**MCH-17**										
*APS_MCH17_1*	GENE-2768_150	4A	1	102.61	3.27	0.0007	3.47	G (−1.90)	–	–
*APS_MCH17_2*	tplb0051b16_1324	4A	49	473.69	3	0.001	3.13	A (0.97)	TraesCS4A02G192700	–
*APS_MCH17_3*	Ra_c27465_564	4B	68	519.26	3.01	0.001	3.17	C (0.98)	TraesCS4B02G253300	Glycoside hydrolase family 65
*APS_MCH17_4*	wsnp_Ku_rep_c72362_72059764	5A	140	694.97	3.25	0.0007	3.34	C (−1.54)	TraesCS5A02G538200	CCR4-NOT transcription complex subunit 1
**SAT-17**										
*APS_SAT17_1*	RAC875_c95364_259	1A	18	1.23	3.25	0.0007	3.67	A (5.99)	TraesCS1A02G002500	Phospholipid-transporting ATPase (P-type ATPase)
*APS_SAT17_2*	Kukri_c13107_100	1A	83	505.51	3.39	0.0006	3.8	A (7.70)	–	–
*APS_SAT17_3*	wsnp_Ku_c15214_23756800	1A	84	503.31	4.35	0.0004	5.08	C (−8.61)	TraesCS1A02G311400	Transcription elongation factor SPT6
*APS_SAT17_4*	wsnp_Ku_c1642_3232242	1A	85	507.08	3.65	0.0006	4.15	C (−8.08)	TraesCS1A02G315600	Peroxisomal leader peptide-processing protease also known as Trypsin domain-containing protein 1
*APS_SAT17_5*	Ku_c1313_1673	1A	86	513.66	3.25	0.0007	3.63	C (−7.73)	TraesCS1A02G323000	P-loop containing nucleoside triphosphate hydrolase (the P-loop NTPase)
*APS_SAT17_6*	wsnp_JD_c6544_7697578	1A	87	514.14	4.16	0.0004	4.85	A (−8.46)	TraesCS1A02G323600	P-loop containing nucleoside triphosphate hydrolase
*APS_SAT17_7*	RAC875_c80876_67	1A	88	515.55	4.3	0.0004	5.03	G (8.68)	TraesCS1A02G324800	Transcription elongation factor SPT5
*APS_SAT17_8*	Excalibur_c44668_382	1A	90	516.45	3.81	0.0005	4.41	A (8.11)	TraesCS1A02G325700	–
*APS_SAT17_9*	RAC875_c45115_509	1A	92	517.49	3.47	0.0006	4.02	A (7.94)	–	–
*APS_SAT17_10*	Kukri_c18017_1696	1A	94	520.34	4	0.0005	4.69	A (−8.45)	TraesCS1A02G331200	Tr-type G domain-containing protein (P-loop GTPases)
*APS_SAT17_11*	RFL_Contig5277_1141	2A	97	771.51	3.4	0.0006	3.8	C (−12.48)	TraesCS2A02G577600	3-Isopropylmalate dehydrogenase
*APS_SAT17_12*	RAC875_c83401_115	4A	110	650.38	3.75	0.0005	4.46	C (6.92)	TraesCS4A02G376100	Cytochrome P450 enzymes
*APS_SAT17_13*	GENE-3758_627	6B	80	665.74	4.23	0.0004	4.85	A (16.91)	TraesCS6B02G391500	T-complex protein 1 subunit delta
*APS_SAT17_14*	BS00037462_51	6B	83	665.51	4.24	0.0004	4.87	C (16.94)	TraesCS6B02G391200	–
*APS_SAT17_15*	BobWhite_c13202_399	6B	122	720.51	3.48	0.0006	3.93	C (7.88)	TraesCS6B02G473000	Thimet oligopeptidases (TOP)
*APS_SAT17_16*	Ku_c15750_761	7A	127	130.19	3.06	0.0009	3.33	C (−8.37)	–	–
**SAT-18**										
*APS_SAT18_1*	BobWhite_rep_c66957_84	7B	70	213.09	3.62	0.0006	4.11	C (−13.44)	–	–
*APS_SAT18_2*	RFL_Contig2540_306	7B	71	185.39	3.41	0.0006	3.88	C (13.18)	TraesCS7B02G144200	–
*APS_SAT18_3*	BS00095819_51	7B	72	190.8	3.42	0.0006	3.88	C (−13.20)	TraesCS7B02G146300	RRM domain-containing protein

Chr, chromosome; Pos, position in cM and Mb. *FDR-adjusted p value (<0.05).

The putative CG associated with the significant SNP markers for STB resistance at both SRT and APS, which were annotated using BLAST search, have been reported in Tables 2, 3. Most of the detected QTL were located in genomic regions enriched with functional domains involved in diverse cellular processes including host plant defense. In total, we have reported 21 CG (7 at SRT and 14 at APS).

## Discussion

In this study, SAMP consisting of 377 ABLs from spring bread wheat breeding program of ICARDA was screened with two virulent *Z. tritici* isolates from Morocco at the seedling stage under controlled conditions, and at the adult plant stage for two cropping seasons at two different field locations in Morocco. Furthermore, GWAM analysis with ∼15k SNP markers detected novel QTL from diverse genomic regions conditioning resistance to STB. To our knowledge, this is the first study on GWAM of STB resistance in spring bread wheat from Morocco.

Wheat–*Zymoseptoria tritici* pathosystem follows the gene-for-gene model (Brading et al., 2002) which states that for each resistant gene in the plant, there is a corresponding avirulence gene in the pathogen (Flor, 1971). In our study, both isolates varied in their virulence spectrum toward 377 wheat genotypes tested. About 45 ABLs were resistant against SAT-2, while 32 ABLs were resistant against 71-R3 STB isolate. The presence of only 13 (20%) resistant ABLs shared by both STB isolates and 51 (80%) ABLs being unique to each STB isolate indicates that both STB isolates differ in their virulence spectrum (Figure 1B). This was further corroborated by race analysis and LSD analysis (Louriki et al., unpublished data). Both STB isolates, SAT-2 and 71-R3, were tested on a set of differentials and different *Stb* genes (*Stb 2*, *Stb 3*, *Stb 4*, and *Stb 16*) were found to be effective against them (Louriki et al., unpublished data). Possibly, these genotypes which were resistant against these isolates may possess new but uncharacterized or known *Stb* genes individually or in combination.

Reactions of wheat genotypes to *Z. tritici* at the adult plant and seedling stages were different. The evaluation of adult plant stage resistance revealed that 5 lines (SAMP-108, 262, 283, 304, and 356) that exhibited resistance at the adult plant stage were susceptible at the seedling stage to both isolates. These lines may possess genes with quantitative effects which are expressed at the adult plant stage but not at the seedling stage. It has been shown that the STB resistance gene *Stb17* has quantitative effect, and it was expressed in adult plant stage but not in seedling stage (Tabib Ghaffary et al., 2011). Unlike *Stb17*, the qualitative gene *Stb15* is not associated with field resistance (Brown et al., 2015).

At Sidi Allal Tazi, the number of resistant genotypes was different during the two cropping seasons (Figure 2 and Supplementary Table 4). In 2017, there were more resistant genotypes (177 lines) than in 2018 (41 lines). This can be attributed to contrasting weather conditions during 2017 and 2018 (Supplementary Table 5). A high disease pressure of *Z. tritici* in 2018 can be explained by humid conditions with abundant rainfall and lower temperature. In addition to weather conditions, several factors such as sowing date, differences in phenology between locations, artificial inoculation, and the amount of initial inoculum may cause the variation in infection response to STB in the field (Thomas et al., 1989; Shaw and Royle, 1993). Interestingly, 10 genotypes (Table 1) showed resistance at both locations (SAT and MCH) during the two cropping seasons. Among those 10 genotypes, two were of synthetic origin and 8 genotypes were of elite origin (Table 1). Interestingly, one genotype (SAMP-275) showed resistance to both STB isolates at seedling as well as at the adult plant stage. Arraiano and Brown (2006) reported that when a line is resistant to different isolates, it may carry isolate specific resistance, or possess partial resistance or even a combination of different *Stb* resistance genes. Currently, we are investigating our hypothesis through forward genetics in a bi-parental double haploid mapping population.

Marker distribution showed that the D genome had fewer polymorphic markers (12%) compared with A (37%) and B (51%) genomes, suggesting a lower level of genetic diversity and recombination in the D genome. According to the evolutionary history of hexaploid wheat (*Triticum aestivum*) with three genomes (AABBDD), tetraploid wild emmer *Triticum dicoccoides* (AABB) was produced when diploid wheat (*T. urartu*, genome AA) hybridized with the goat grass (*Aegilops speltoides*, genome BB) 300,000–500,000 years ago. However, the D genome was recently introduced into *T. dicoccoides* (AABB) upon hybridization with another goat grass (*Aegilops tauschii*, genome DD) about 9,000 years ago (Dvorak and Akhunov, 2005; Peng et al., 2011). Therefore, a much greater degree of genetic diversity and recombination exists between AABB genomes than between AABB and DD genomes in the present soft wheat which could explain a lower number of SNP markers on the D genome in our study as reported previously (Dubcovsky and Dvorak, 2007; Nielsen et al., 2014; Vagndorf et al., 2017; Ayana et al., 2018).

**FIGURE 6:**
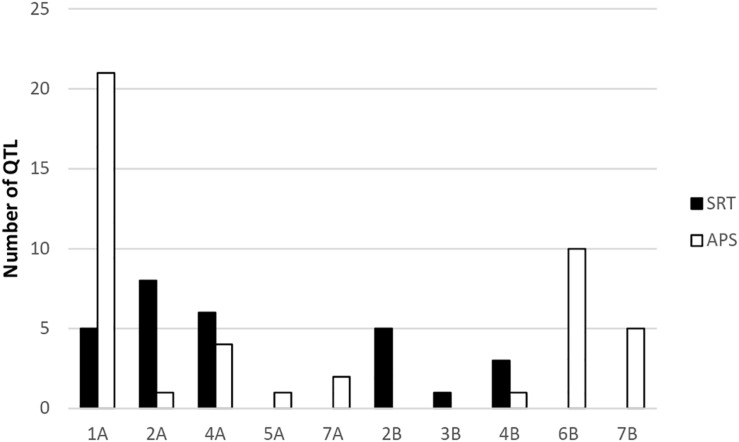
Frequency distribution of QTL at SRT and APS.

Our genome-wide scan has detected 14 SRT and 23 APS QTL associated with STB resistance on diverse chromosomes (Figure 5). Fourteen QTL were associated with STB resistance at the seedling stage to both tested isolates (Table 3). These QTL are located on six chromosomes: 1A (three), 2A (two), 4A (three), 2B (three), 3B (one), and 4B (two). At adult plant stage, 23 QTL were associated with eight chromosomes: 1A (ten), 2A (one), 4A (three), 5A (one), 7A (one), 4B (one), 6B (three), and 7B (three) (Table 4). QTL for both APS and SRT were found on chromosomes 1A, 2A, 4A, and 4B, whereas QTL on chromosomes 2B and 3B were associated exclusively with SRT (Figure 6 and Table 3). Similarly, QTL on chromosomes 5A, 7A, 6B, and 7B were specific to APS (Figure 6 and Table 4). Of all the 14 SRT QTL detected, the 6 SNP marker alleles associated with 6 QTL had negative allele effect which means that they decrease the disease severity. Allele C of SNP marker RAC875_c17787_274 associated with the QTL *SRT_SAT2_3* (92 cM) on chromosome 2A had the lowest allele effect (-9.39), whereas allele A of SNP marker wsnp_Ex_c5072_9006666 associated with the QTL *SRT_SAT2_7* (164 cM) on chromosome 4A had the highest allele effect (-14.08) for reducing the pycnidia development (Table 3). Similarly, of the 23 APS QTL, 11 SNP marker alleles of the associated QTL had negative allele effect (Table 4). Allele C of SNP marker wsnp_Ku_rep_c72362_72059764 associated with QTL *APS_MCH17_4* (140 cM) on chromosome 5A had the lowest allele effect (-1.54), whereas allele C of SNP marker BobWhite_rep_c66957_84 associated with QTL *APS_SAT18_1* (70 cM) on chromosome 7B had the highest allele effect (-13.44) for reducing the STB disease severity in the field. Some of these SNP markers will be good candidates for MAS in STB resistance breeding efforts.

Some identified QTL may coincide with mapping locations of known STB resistance genes. For example, peak markers of three QTL *SRT_71-R3_2* (143 cM), *SRT_71-R3_3* (144 cM), and *SRT_71-R3_4* (145 cM) on chromosome 2B were identified at similar chromosomal region as STB resistance gene *Stb9*. The SSR marker Xwmc317 associated with *Stb9* gene is located on the long arm of chromosome 2B (Brown et al., 2015). In our study, the identified QTL are located on the long arm of the chromosome 2B as well and were found in close physical proximity (4–8 Mb) to SSR Xwmc317, which is linked to the *Stb9* gene. This gene was reported to be active at the seedling stage and these markers could be associated to *Stb9.* In addition, peak markers of two SRT QTL (*SRT_SAT2_6* and *SRT_SAT2_7*) were positioned on the long arm of chromosome 4A. The known *Stb* resistance genes on this chromosome are *Stb7* and *Stb12*. Previous studies mapped these two *Stb* genes on the long arm of the chromosome 4A (McCartney et al., 2003; Brown et al., 2015). Based on the physical map positions, QTL were in close physical proximity (3–4 Mb) to SSR markers Xwmc313 and (9–10 Mb) to Xwmc219, which are linked to *Stb7* and *Stb12*, respectively.

On chromosome 5A, we detected an APS QTL (*APS_MCH17_4*) whose physical position (694 Mb) is in the region of a previously published QTL (*QTL9*) by Goudemand et al. (2013), conferring resistance to STB. However, the *QTL9* was associated with STB resistance at the seedling stage while in our study the QTL (*APS_MCH17_4*) was associated with the adult plant resistance. On chromosome 3B, a peak marker of an SRT QTL (*SRT_SAT2_5*) was located not far from SSR marker Xwmc500 (about 11 Mb), which is linked to *Stb14* (Cowling, 2006). Thus, this peak marker (IAAV6442) could be associated to this *R* gene.

Three APS QTL *APS_SAT18_1* (70 cM), *APS_SAT18_2* (71 cM), and *APS_SAT18_3* (72 cM) were identified in close proximity of *Stb8* or *Stb13* on chromosome 7B. The SSR markers linked to these *Stb* genes were located on the long arm of chromosome 7B (Brown et al., 2015). However, in this study, the peak markers on 7B were located on the short arm of the chromosome. No QTL coincided in the genomic region of *Stb6* (3AS) which is the only qualitative gene for STB resistance which has been cloned and functionally characterized (Saintenac et al., 2018). We found three APS QTL on chromosome 6B [*APS_SAT17_13*(80 cM), *APS_SAT17_14* (83 cM), *APS_SAT17_15* (122 cM)] and one SRT QTL on chromosome 3B [SRT_SAT2_5 (30 cM)]. Similarly, Brown et al. (2015) reported the involvement of QTL on chromosomes 3B, 6B, and 7D especially in quantitative resistance to STB. To our knowledge, none of the 21 known *Stb* genes have been mapped on chromosomes 1A, 2A, and 4B. But we have found 3 SRT and 10 APS QTL on chromosome 1A, accounting for 13.7 and 43.3% of phenotypic variation, respectively (Tables 3 and 4). These QTL are novel and have never been associated with any known STB resistance genes. Some of these newly identified QTL are good candidates for MAS for STB resistance breeding program of Morocco. SRT and APS QTL on chromosome 2A (*SRT_SAT2_3*, 92 cM; *SRT_SAT2_4*, 104 cM; *APS_SAT17_11*, 97 cM) and 4B (*SRT_71-R3_6*, 2 cM; *SRT_SAT2_8*, 89 cM; *APS_MCH17_3*, 68 cM) are also regarded as novel. It is noteworthy that a QTL *SRT_71-R3_4* detected on chromosome 2B at SRT is overlapping with another QTL previously reported by Muqaddasi et al. (2019) at APS at the same position (145 cM).

To understand the relation between significant SNP markers and STB resistance, we identified and reviewed the annotation of putative CG associated with them. On chromosome 1A, a peroxisomal leader peptide-processing protease, also known as Trypsin domain-containing protein-1, was associated with the peak marker (wsnp_Ku_c1642_3232242) of QTL *APS_SAT17_4* at 85 cM. It has been well documented that plant peroxisomal proteins play an essential role in different metabolic functions and plant responses to abiotic and biotic stresses (Reumann et al., 2016). In addition, we found that chromosome 1A harbored a P-loop ATPase domain protein (Peak marker Ku_c1313_1673 of QTL *APS_SAT17_5* at 86 cM). It is well known that most of the *R* genes identified to date have the p-loop ATPase domain, which has been involved in biotic stress response (Arya and Acharya, 2017; Sathuvalli et al., 2017). In the same genomic region, a phospholipid-transporting ATPase (P-type ATPase) was found to be associated with STB resistance in the present study. In rice, Gilbert et al. (2006) demonstrated that a P-type ATPase-encoding gene, *MgAPT2*, is required for a rapid induction of host defense responses to rice blast pathogen *Magnaporthe grisea*. On chromosome 2A, the peak marker (Ra_c22880_760) of SRT QTL *SRT_SAT2_4* (104 cM) lays within a gene encoding alpha amylase inhibitor. In plants, these substances are well known for their involvement in host plant defense against pests like *Callosobruchus maculatus* and *Zabrotes subfasciatus* (Franco et al., 2002; Wisessing and Choowongkomon, 2012) and diverse pathogens like *Aspergillus flavus* (Rajasekaran et al., 2019).

On chromosome 4A, the peak marker wsnp_Ex_c5072_9006666 of QTL *SRT_SAT2_7* (164 cM) lays within a gene encoding SAM-dependant MTase DRM-type. Plant DRM (Domains Rearranged Methylase) enzymes are required for DNA methylation, which is a conserved mechanism of epigenetic gene regulation and genome defense (Zhong et al., 2015). Several studies have reported the role of DNA methylation in plant immunity. For example, the decrease in DNA methylation enhanced wheat progenitor *A. tauschii* resistance to *Blumeria graminis* f. sp. *tritici* (Geng et al., 2019). Furthermore, in *Arabidopsis thaliana*, it has been reported that the defense responses to *Pseudomonas syringae* (Yu et al., 2013) and *Fusarium oxysporum* (Le et al., 2014) were enhanced by mutations in DNA methylation. In this genomic region, the peak marker RAC875_c83401_115 associated with QTL *APS_SAT17_12* (110 cM), lays within a gene encoding Cytochrome P450 enzyme, which is important for the biosynthesis of several compounds such as phytohormones and other defense molecules (Pandian et al., 2020).

The peak markers Excalibur_c5438_274 and BobWhite_c10520_1517 of two SRT QTL (*SRT_71-R3_2*, 143 cM; *SRT_71-R3_3*, 144 cM) on chromosome 2B encompass genes *TraesCS2B02G588900* and *TraesCS2B02G587900* which encode for class 2 dihydroorotate dehydrogenase (DHODH) and the STT3 subunit, respectively. In *Arabidopsis*, the STT3 subunit was found to be essential for plant development and survival (Koiwa et al., 2003). Likewise, on chromosome 3B, WD40-repeats protein was associated with the peak marker IAAV6442 of QTL *SRT_SAT2_5* (30 cM). WD-40 repeats proteins are ubiquitous in all eukaryotes and have been implicated in diverse functions like cell wall formation and plant immunity (Guerriero et al., 2015).

On chromosome 4B, the peak marker Tdurum_contig60051_838 (*SRT_71-R3_6*, 2 cM) lays within a gene encoding pyridoxal phosphate-dependent transferase. Pyridoxal phosphate (PLP), the active form of vitamin B_6_, was shown to be an excellent antioxidant and played an important role in plant development and stress tolerance (Czégény et al., 2019). In addition, we found that the peak marker associated with the QTL *APS_MCH17_3* (68 cM) on chromosome 4B was associated with glycoside hydrolase 65 gene which belongs to the glycoside hydrolases (GHs) family. The GH65 family members have been reported in *A. thaliana*, *Oryza sativa*, and *Populus trichocarpa*. These enzymes are involved in diverse processes, including plant defense response (Tyler et al., 2010). Similarly, the peak marker BobWhite_c13202_399 of QTL (*APS_SAT17_15*) on chromosome 6B (122 cM) annotated Thimet oligopeptidases family (TOP). In plants, these metallopeptidases are targets for salicylic acid (SA) and seem to play a role in SA-dependent innate immunity (Wang et al., 2014). Besides this marker on chromosome 6B, another marker, namely GENE-3758_627 of *APS_SAT17_13* (80 cM) QTL, laid within a gene encoding T-complex protein 1 subunit delta (TCP-1-delta/Chaperonin CCT4), which belongs to the group II chaperonin complex called CCT. These molecular chaperones have been known in preventing incorrect folding and formation of non-functional structures (Mori et al., 1992). The remaining QTL on chromosomes 1A, 5A, and 7B were found to be transcription regulation factors.

Heading date is an important trait that may affect STB resistance depending upon the set of genotypes used (Simón et al., 2004; Arraiano et al., 2009). In SAMP, the mean HD of 91 ± 4.0 was recorded. Furthermore, we observed that STB resistance in SAMP is not linked to the HD as reported earlier by Arama et al. (1999) and Simón et al. (2005). The genomic regions associated with STB resistance were located at different genetic positions compared with HD QTL identified in SAMP (unpublished data) and in previously reported QTL for HD (Risser et al., 2011; Zanke et al., 2014; Gerard et al., 2017; Huang et al., 2018; Li et al., 2019; Sheoran et al., 2019). Using HD as a covariate, QTL for heading date were mapped on chromosome 2A (6 and 17 cM), whereas STB QTL was mapped on chromosome 2A; *SRT_SAT2_3*, *SRT_SAT2_4*, and *APS_SAT17_11* were located far apart on 92, 104, and 97 cM, respectively (Supplementary Figure 4). Similarly, HD QTL on chromosome 2B (79 cM) is far apart from STB QTL *SRT_71-R3_2* (143 cM), *SRT_71-R3_3* (144 cM), and *SRT_71-R3_4* (145 cM). There was no influence of heading date on the expression of STB resistance as 5/6 QTL detected on chromosomes 2A and 2B are SRT QTL except for APS QTL (*APS_SAT17_11*) which was 80–91 cM away from HD QTL detected on 2A.

We have used 377 elite spring bread wheat genotypes for this association mapping study. For GWASs, many authors have used population sizes of 167 and above. From such studies, gene annotations have been carried out. The putative CG underlying the identified STB QTL should be further validated using biparental mapping populations after genotyping with high-density markers. In addition, reverse genetic approach could be employed to functionally characterize the cloned putative CG.

Jighly et al. (2015) genotyped 200 ICARDA wheat genotypes with 2,688 DArT and 4,252 SNP markers to identify QTL associated with stripe rust resistance in wheat. Furthermore, Tadesse et al. (2014) genotyped 167 facultative/winter elite wheat genotypes with 3,051 DArT markers and reported 10 markers associated with stripe rust resistance.

In summary, significant differences were observed among the advanced elite spring bread wheat breeding lines for Septoria resistance both at seedling and at the adult plant stages. This is one of the most comprehensive studies published on GWAM of STB resistance from North Africa. These genotypes will be distributed to the national wheat breeding programs of the world through the regular ICARDA’s international nursery system for potential direct release or to be used as parents in hybridization schemes with elite cultivars. We also recommend the significant markers reported in this study to be validated before using them for marker-assisted selection in the wheat breeding programs. The moderately resistant advance breeding lines could also be used to pyramid genes to achieve a more durable and race non-specific resistance to STB.

## Data Availability Statement

The raw data supporting the conclusions of this article will be made available by the authors, without undue reservation.

## Author Contributions

SL and SR conceived and coordinated the study. SL, SR, and MA-J collected the phenotypic data. SL, SR, and YB performed the statistical and bioinformatics analysis. WT provided genotypic data. SL, SR, SE, AD, AA, and WT reviewed and contributed to the manuscript. All authors contributed to the article and approved the submitted version.

## Conflict of Interest

The authors declare that the research was conducted in the absence of any commercial or financial relationships that could be construed as a potential conflict of interest.

## Footnotes

1 https://cran.r-project.org/

2 https://www.biosearchtech.com/services/genotyping-services/array-genotyping/plant-array-genotyping

3 https://wheat-urgi.versailles.inra.fr/

4 http://www.plants.ensembl.org/Triticum_aestivum/Tools/Blast

## Supplementary Material

Supplementary Figure 1 Quantile–quantile (Q-Q) plots of marker–trait association at SRT stage for STB isolate SAT2 and 71-R3 using GLM+Q model **(A)**, GLM+PCA model **(B)**, MLM + Q + K model **(C)**, and MLM + PCA + K model **(D)**.

Supplementary Figure 2 Quantile–Quantile (Q-Q) plots of marker–trait association at the adult plant stage (APS) at Sidi Allal Tazi during 2017 (SAT-17), 2018 (SAT-18), and at Marchouch during 2017 (MCH-17) using GLM + Q model **(A)**, GLM + PCA model **(B)**, MLM + Q + K model **(C)**, and MLM + PCA + K model **(D)**.

Supplementary Figure 3 Manhattan plots displaying significant MTAs for STB resistance in SAMP bread wheat panel using MLM model in Tassel (v 5.2.53) at seedling (SRT) stage for STB isolates SAT2 **(A)**, 71-R3 **(B)**, and at the adult plant stage (APS) for MCH-17 **(C)**, SAT-17 **(D)**, and SAT-18 **(E)**.

Supplementary Figure 4 Manhattan plot displaying significant MTAs for days to heading (HD) in SAMP bread wheat panel using MLM model in Tassel (v v 5.2.53).

Supplementary Table 1 The list of genotypes included in the SAMP with pedigree information.

Supplementary Table 2 Zymoseptoria tritici isolates used in SRT and APR screening of advance bread wheat breeding lines included in SAMP.

Supplementary Table 3 Infection responses of wheat genotypes at the seedling (STB isolates; SAT-2, 71-R3), at the adult plant stage (MCH-17, SAT-17, SAT-18), and days to heading (HD).

Supplementary Table 4 Frequency distribution of adult plant resistance of SAMP to Septoria tritici blotch at SAT-17, SAT-18, and MCH-17.

Supplementary Table 5 Average monthly weather in Sidi Allal Tazi, Morocco during 2017 and 2018.
