# Supplementary material for: Identification of Resistance Sources and Genome-Wide Association Mapping of Septoria Tritici Blotch Resistance in Spring Bread Wheat Germplasm of ICARDA
Source: Front Plant Sci. 2021 May 25;12:600176. doi: 10.3389/fpls.2021.600176 (PMC8185176; doi:10.3389/fpls.2021.600176)

**Supplementary Figure S2.** Quantile-Quantile (Q-Q) plots of marker-trait association at the adult plant stage (APS) at Sidi Allal Tazi during 2017 (SAT-17), 2018 (SAT-18), and at Marchouch during 2017 (MCH-17) using GLM+Q model (a), GLM+PCA model (b), MLM + Q + K model (c), and MLM + PCA + K model (d).

a) GLM + Q

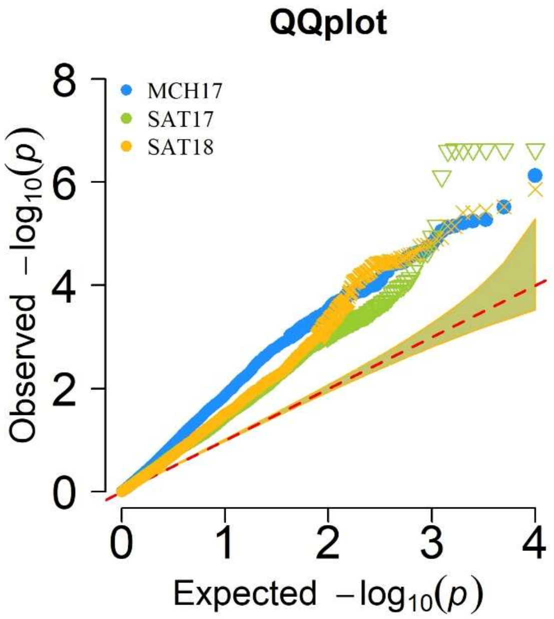


b) GLM + PCA

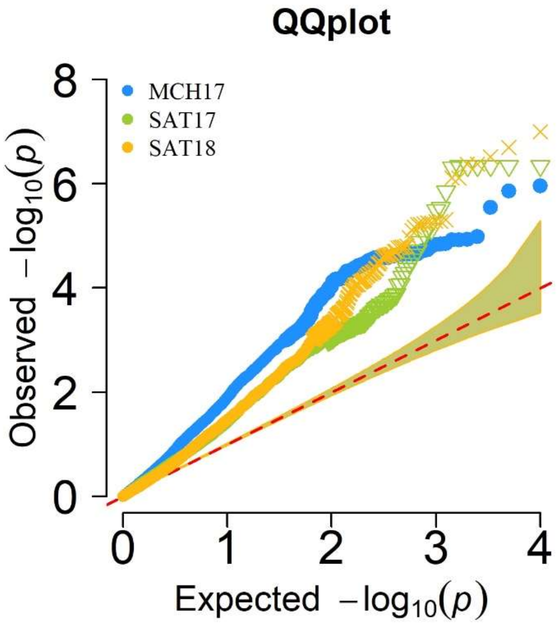

c) MLM + Q + K

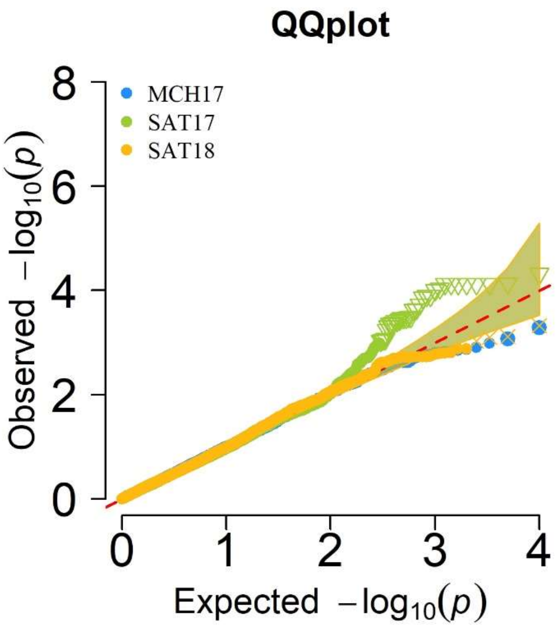


d) MLM + PCA + K

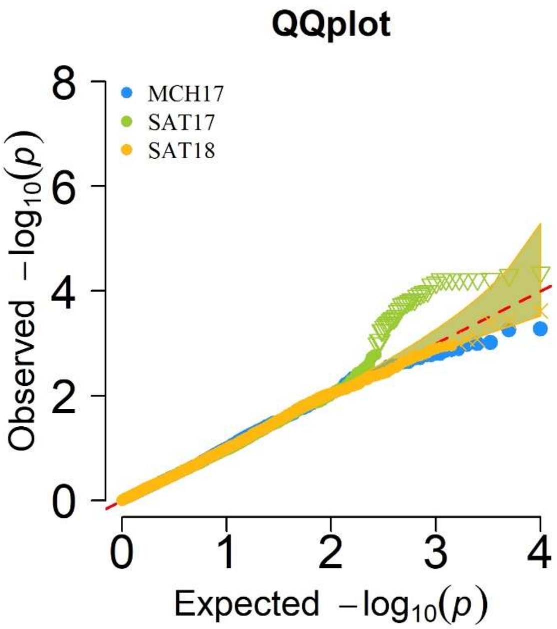

Supplement: Supplementary Figure 2 — Quantile–Quantile (Q-Q) plots of marker–trait association at the adult plant stage (APS) at Sidi Allal Tazi during 2017 (SAT-17), 2018 (SAT-18), and at Marchouch during 2017 (MCH-17) using GLM + Q model (A), GLM + PCA model (B), MLM + Q + K model (C), and MLM + PCA + K model (D). [file Image_2.PDF]
