# Supplementary material for: Identification of Resistance Sources and Genome-Wide Association Mapping of Septoria Tritici Blotch Resistance in Spring Bread Wheat Germplasm of ICARDA
Source: Front Plant Sci. 2021 May 25;12:600176. doi: 10.3389/fpls.2021.600176 (PMC8185176; doi:10.3389/fpls.2021.600176)
Supplement: Supplementary Figure 3 — Manhattan plots displaying significant MTAs for STB resistance in SAMP bread wheat panel using MLM model in Tassel (v 5.2.53) at seedling (SRT) stage for STB isolates SAT2 (A), 71-R3 (B), and at the adult plant stage (APS) for MCH-17 (C), SAT-17 (D), and SAT-18 (E). [file Image_3.PDF]

Supplementary Figure S3. Manhattan plots displaying significant MTAs for STB resistance in SAMP bread wheat panel using MLM model in Tassel (v v 5.2.53) at seedling (SRT) stage for STB isolates SAT2 (a), 71-R3 (b), and at the adult plant stage (APS) for MCH-17 (c), SAT-17 (d), and SAT-18 (e).

a

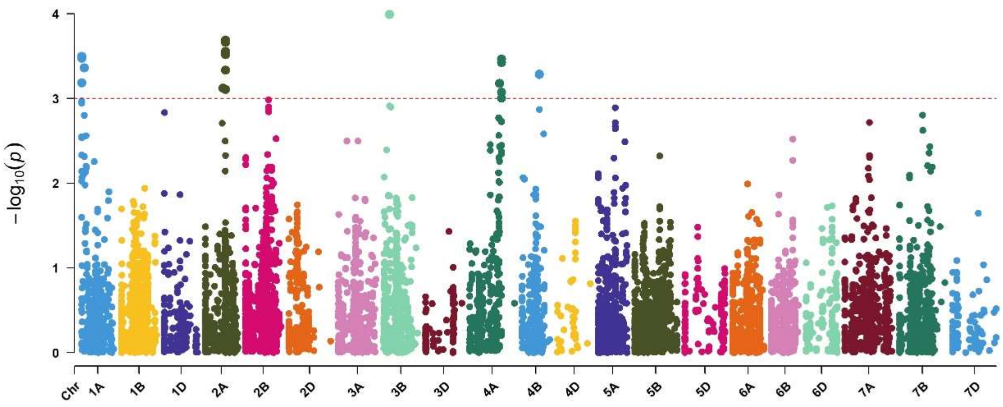

b

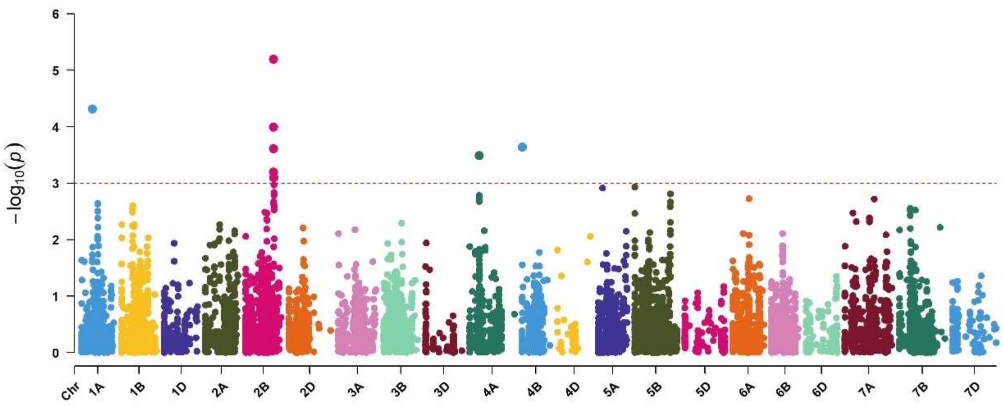

c

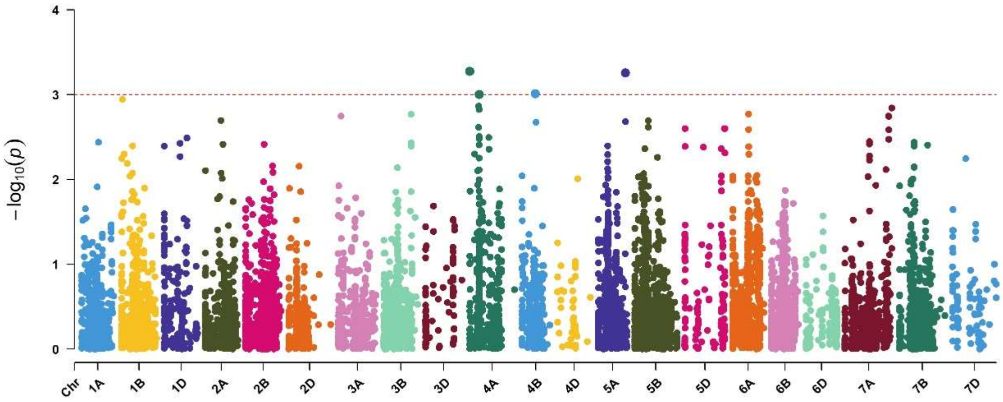

−log10(p)
Chr
1A
1B
1D
2A
2B
2D
3A
3B
3D
4A
4B
4D
5A
5B
5D
6A
6B
6D
7A
7B
7D

d

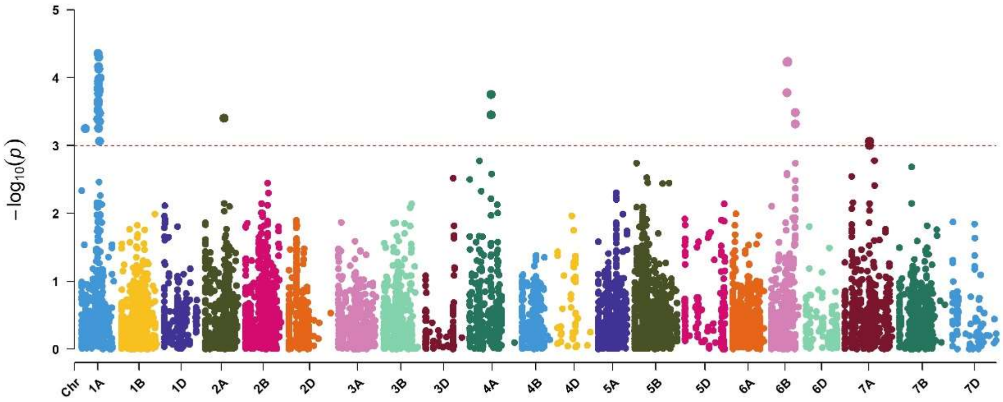

−log10(p)
Chr
1A
1B
1D
2A
2B
2D
3A
3B
3D
4A
4B
4D
5A
5B
5D
6A
6B
6D
7A
7B
7D

e

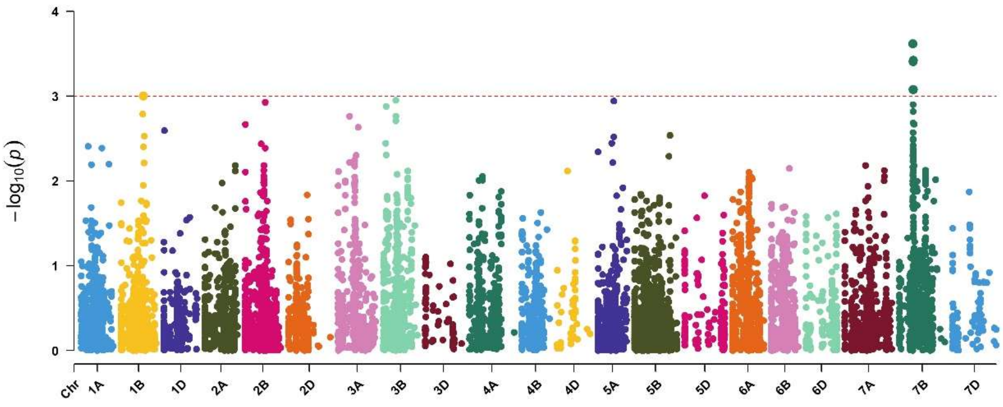

−log10(p)
Chr
1A
1B
1D
2A
2B
2D
3A
3B
3D
4A
4B
4D
5A
5B
5D
6A
6B
6D
7A
7B
7D
