# Supplementary material for: Identification of Resistance Sources and Genome-Wide Association Mapping of Septoria Tritici Blotch Resistance in Spring Bread Wheat Germplasm of ICARDA
Source: Front Plant Sci. 2021 May 25;12:600176. doi: 10.3389/fpls.2021.600176 (PMC8185176; doi:10.3389/fpls.2021.600176)

Supplementary Figure S4. Manhattan plot displaying significant MTAs for days to heading (HD) in SAMP bread wheat panel using MLM model in Tassel (v v 5.2.53).

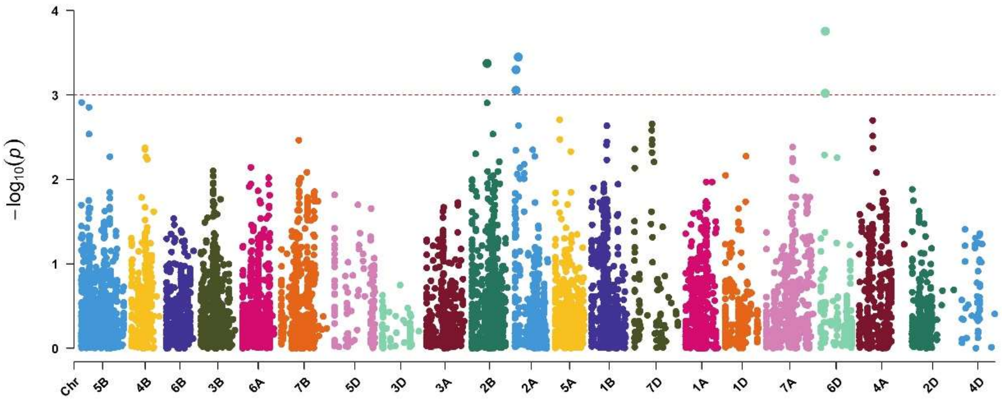

Supplement: Supplementary Figure 4 — Manhattan plot displaying significant MTAs for days to heading (HD) in SAMP bread wheat panel using MLM model in Tassel (v v 5.2.53). [file Image_4.PDF]
